# Abscess of the Sheath of the Flexor Pedis Perforans Tendon

**Published:** 1898-06

**Authors:** W. J. Martin

**Affiliations:** Kankakee, Ill.


### REPORTS OF CASES.

### ABSCESS OF THE SHEATH OF THE FLEXOR PEDIS PERFORANS TENDON.

By W. J. Martin, V.S.,
KANKAKEE, ILL.

During the past winter the patient, a black mare, aged twelve years, used for farm purposes, was kicked by another horse upon the internal posterior surface of the right hock-joint, midway between the summit of the calcaneum and its base and directly over the tendon of the flexor pedis perforans muscle. The calk of the shoe which inflicted the injury being very blunt, but a slight wound was made. The owner considering the wound of not much importance, did nothing but apply some simple healing-salve, expecting that in a few days the wound would heal. After the expiration of several weeks, the injury not being inclined to heal and the mare being slightly lame, I was requested to visit her.

On examination the joint was found to be but slightly swollen upon its internal surface; at the point of the injury a smooth fistulous opening, surrounded by a rim of fungoid tissue, was seen. The external surface of the joint was somewhat thickened and indurated. As near as I could learn, nothing resembling synovial fluid had been discharged from the fistulous opening. The discharge consisted, the owner stated, of a small amount of bloody water. Lameness was not very marked; in standing the mare seemed to bear considerable weight upon the limb. The physical condition of the animal was good, the appetite not being in the least impaired.

Owing to the length of time since the infliction of the injury, a guarded prognosis was given, pointing out to the owner the probability of acute arthritis or synovitis setting in at any time and destroying the structure of the joint.

The treatment adopted was of the mild and expectant order, such as absolute rest in a stall, the inunction of camphorated oil around the joint, and directly over the fistulous opening was smeared a mild compound ointment of iodine. Under this method of treatment the mare seemed to improve rapidly, and after the expiration of three weeks nearly all lameness and swelling of the joint had disappeared. The fistulous tract, however, still remained open and discharged but a small quantity of a clear watery fluid.

So confident had the owner become that the mare was about well that he began to give her walking exercise and to use oxide-of-zinc ointment to heal the "pipe" in the joint. After using this ointment for a week or ten days the joint suddenly began to swell and the mare became very lame. The owner then placed the mare in a sleigh and brought her to my infirmary, a distance of fourteen miles.

Upon her arrival the leg presented the following condition: The internal surface of the hock-joint over the course of the flexor pedis perforans muscle was enormously swollen and indurated; this swelling surmounted the point of the calcaneum, forming an exaggerated capped hock. The external aspect of the hock was also enlarged and indurated. The anterior surface of the hock was not enlarged save for a slight distention of fluid at the seat of bog-spavin. Otherwise this part of the joint was in a perfectly normal condition. The old fistulous opening was now much larger than formerly, and from it was discharged a considerable amount of bloody watery fluid. The lameness was most severe, the animal barely placing the foot to the ground for a moment, then quickly catch it up again. The appetite was still good, and her bodily condition was fair.

The joint now presented nearly every indication of being the seat of extensive articular arthritis, yet some very essential symptoms of this affection were wanting, such as the absence of all swelling and inflammatory action in the anterior part of the joint. Also the excellent physical condition in the mare showed the absence of all irritative fever, etc.

I must confess that in all my experience with diseases of the hock-joint, none had ever perplexed me in trying to locate the exact nature and extent of the disease as this case had done. All the literature at my command was ransacked for information of a similar case, but in vain.

The treatment adopted was of the most energetic kind; the fistulous opening was enlarged and thoroughly curetted; Lugol's solution of iodine was then injected into the cavity; the entire joint was bathed with camphorated oil, then swathed in cotton and a roller bandage applied. This method of treatment was kept up, supplemented from time to time by the application of severe blisters over the diseased area. Iodide of potassium in drachm doses was given in the drinking-water three times a day. Under this form of medication the old fistulous opening healed, as did a new one that formed and broke just above the seat of the old one. This treatment was continued for a month. The enlargement on the internal surface of the joint had become somewhat smaller, but the lameness still remained. After the expiration of another two weeks, all hope of curing the severe lameness which still persisted, the animal was ordered to be destroyed.

*Post-mortem.* At a point about three inches below the fleshy belly of the flexor pedis perforans muscle the tendinous seat of the muscle was found to be greatly distended with a reddish-colored, watery fluid, in which were floating particles of the broken-down débris of the enveloping sheath and adjacent tissue. The pouch or cavity was almost exactly six inches in length, and included the entire surrounding sheath of the tendon. The inner surface of the trochlea of the astragalus was stained a dark-blue color; the capsular ligament of the hock was not diseased; the synovial fluid was perfectly normal in appearance. The connective tissue on the external part of the joint was thickened and indurated. The dark color of the trochlea of the astragalus, no doubt, was due to the permeation of the reddish fluid through the intervening tissues by osmosis.

Had the true nature of the injury been detected during life, and had the treatment been that of aspiration of the fluid in the sheath, or, better still, if the entire sheath of the tendon had been laid open the entire length of the diseased area and then dressed antiseptically, a good recovery might have been made.

